# How Do Stakeholders Make Decisions About AI and Novel Technologies for the Healthcare of Older Adults?

**DOI:** 10.1093/geroni/igaf122.381

**Published:** 2025-12-31

**Authors:** Nancy Schoenborn, Zhang Zhang, Ashida Sato, Thomas Cudjoe

**Affiliations:** Johns Hopkins University School of Medicine, Baltimore, Maryland, United States; Johns Hopkins Bloomberg School of Public Health, Baltimore, Maryland, United States; University of Iowa College of Public Health Department of Community and Behavioral Health, Iowa City, Iowa, United States; Johns Hopkins School of Medicine, Baltimore, Maryland, United States

## Abstract

Artificial intelligence (AI) and novel technologies offer tremendous potential for improving the health of older adults, but how key stakeholders make decisions about which technology to finance and adopt is not well understood. We used qualitative semi-structured interviews to explore how older adults, care partners, clinicians, and insurance payers decide whether to adopt and how investors decide whether to finance new AI technology. Participants included 15 older adults or care partners, 15 clinicians, 8 payers, and 5 investors. Interviews were analyzed using qualitative thematic analysis. All key stakeholders considered cost, usability, and added value, but each group had different perspectives. Older adults and care partners emphasized out-of-pocket costs and the product’s user-friendliness for older adults. Clinicians were concerned about both patient and health system costs and considered usability when fitting into existing workflow. Payers considered the cost and added value from a population health perspective, including the frequency and cost of the health events (e.g., falls) that the technology aims to impact, the revenue stream (e.g., insurance coverage), and the cost of implementation and maintenance. Investors considered cost in terms of return on investment and considered competing products, market demand size, and the project team in decisions. In addition to end-user cost and usability, multiple other factors influence the likelihood that new AI technologies will become available for the healthcare of older adults. Ensuring engagement to raise awareness about key decisional factors and aligning incentives to motivate impactful products for the end-users is important.

